# 1-{2-[(2,4-Dichloro­benzyl­idene)amino]eth­yl}-3-methyl­imidazolium hexa­fluoro­phosphate

**DOI:** 10.1107/S1600536809045115

**Published:** 2009-11-04

**Authors:** Jie Liu, Bin Li, Yi-Qun Li, Wen-Jie Zheng

**Affiliations:** a Department of Chemistry, Jinan University, Guangzhou 510632, People’s Republic of China

## Abstract

In the title Schiff base compound, C_13_H_14_Cl_2_N_3_^+^·PF_6_^−^, the dihedral angle between the aromatic ring and imidazole ring in the cation is 6.10 (2)°. Inter­molecular C—H⋯F hydrogen-bonding inter­actions and π–π stacking inter­actions [centoid–centroid distance = 3.7203 (12) Å] help stabilize the crystal packing.

## Related literature

For bound-length data, see: Allen *et al.* (1987 ▶). For related structures, see: Pradeep (2005 ▶); Li *et al.* (2009 ▶). For ionic liquids and their applications, see: Wasserscheid & Keim (2000 ▶); Singh & Sekhon (2005 ▶); Noda & Watanabe (2000 ▶).
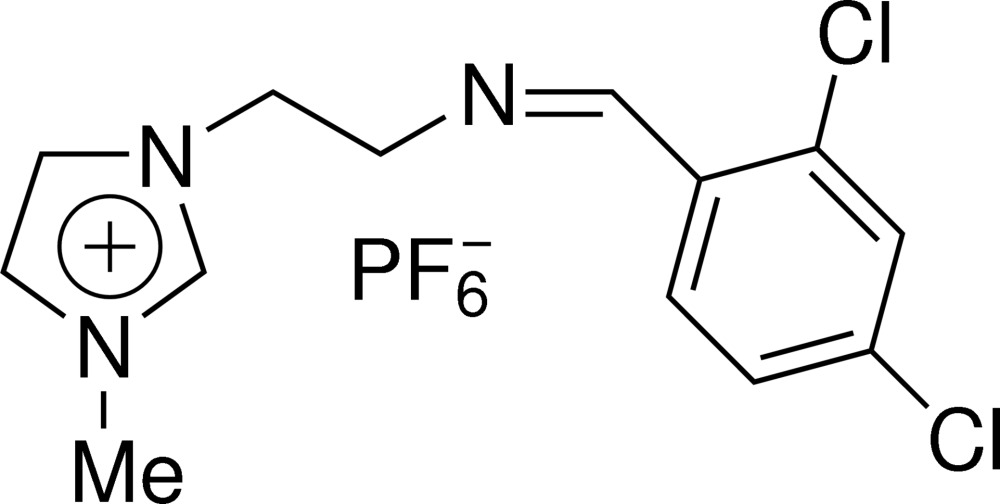


## Experimental

### 

#### Crystal data

C_13_H_14_Cl_2_N_3_^+^·PF_6_^−^
*M* *_r_* = 428.14
Triclinic, 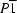

*a* = 8.3465 (13) Å
*b* = 10.1419 (16) Å
*c* = 11.0310 (17) Å
α = 78.899 (2)°
β = 76.523 (2)°
γ = 67.834 (2)°
*V* = 835.3 (2) Å^3^
*Z* = 2
Mo *K*α radiation
μ = 0.55 mm^−1^
*T* = 173 K
0.32 × 0.24 × 0.21 mm

#### Data collection

Bruker SMART CCD area-detector diffractometer
Absorption correction: multi-scan (*SADABS*; Sheldrick, 1996 ▶) *T*_min_ = 0.844, *T*_max_ = 0.894
3566 measured reflections
3566 independent reflections
3151 reflections with *I* > 2σ(*I*)

#### Refinement

*R*[*F* ^2^ > 2σ(*F* ^2^)] = 0.031
*wR*(*F* ^2^) = 0.093
*S* = 1.10
3566 reflections
227 parameters
H-atom parameters constrained
Δρ_max_ = 0.25 e Å^−3^
Δρ_min_ = −0.34 e Å^−3^

### 

Data collection: *SMART* (Bruker, 2002 ▶); cell refinement: *SAINT* (Bruker, 2002 ▶); data reduction: *SAINT*; program(s) used to solve structure: *SHELXS97* (Sheldrick, 2008 ▶); program(s) used to refine structure: *SHELXL97* (Sheldrick, 2008 ▶); molecular graphics: *SHELXTL* (Sheldrick, 2008 ▶); software used to prepare material for publication: *SHELXTL*.

## Supplementary Material

Crystal structure: contains datablocks global, I. DOI: 10.1107/S1600536809045115/jj2012sup1.cif

Structure factors: contains datablocks I. DOI: 10.1107/S1600536809045115/jj2012Isup2.hkl

Additional supplementary materials:  crystallographic information; 3D view; checkCIF report

## Figures and Tables

**Table 1:** Hydrogen-bond geometry (Å, °)

*D*—H⋯*A*	*D*—H	H⋯*A*	*D*⋯*A*	*D*—H⋯*A*
C5—H5⋯F3	0.95	2.51	3.324 (2)	143
C10—H10⋯F3^i^	0.95	2.33	3.203 (2)	152
C10—H10⋯F5^i^	0.95	2.54	3.373 (2)	147
C11—H11⋯F6	0.95	2.46	3.275 (2)	143
C12—H12⋯F2	0.95	2.48	3.239 (2)	137
C13—H13*C*⋯F5^ii^	0.98	2.54	3.464 (2)	158

Symmetry codes: (i) 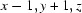; (ii) 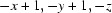.

**Fig. 1.**
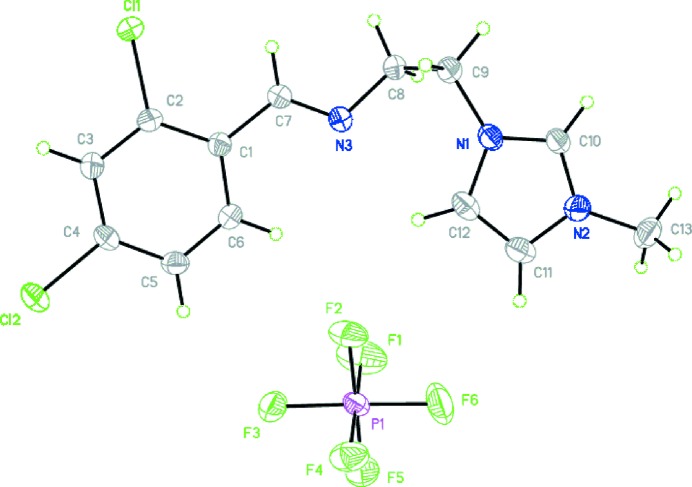
The molecular structure of the title compound in (I) showing the atom numbering Scheme. Displacement ellipsoids are drawn at the 50% probability level.

**Fig. 2.**
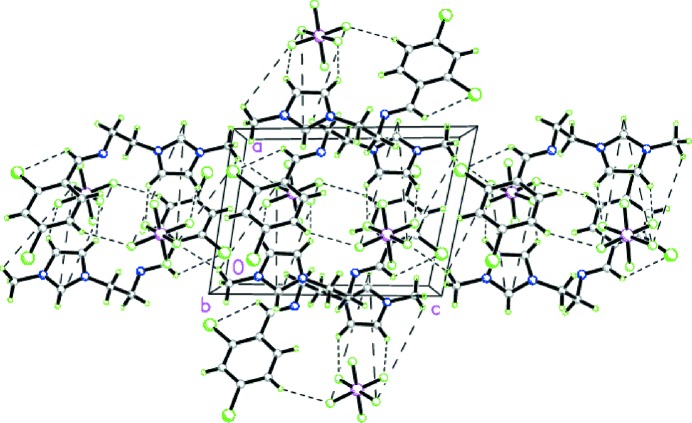
Packing diagram of (I) viewed down the b axis. Weak C–H···Cl intramolecular and C–H···F intermolecular hydrogen bonding interactions are shown as dashed lines.

## supplementary crystallographic information

### Comment

Ionic liquids are attracting much interest in many fields of chemistry and industry, due to their potential as green solvents for a wide range of applications in synthesis, catalysis, electrochemistry, and liquid-liquid extractions (Wasserscheid *et al.*, 2000; Singh, 2005; Noda, 2000). Schiff base compounds are one of most prevalent mixed-donor ligands in the field of coordination chemistry (Li *et al.*, 2009). As part of our program aimed at developing a novel functionalized ionic liquid, we now report the crystal structure of a novel ionic liquid-supported Schiff base (I).

The asymmetric unit of the title compond, (I), a Schiff base derived ionic liquid, is comprised of an organic cation and a PF_6_ counter anion, Fig. 1. Bond lengths and angels are generally within normal ranges (Allen *et al.*, 1987). The dihedral angle between the mean planes of the imidazole and benzene rings in the cation is 6.10°. The crystal structure exhibits weak C–H···Cl intramolecular and C–H···F intermolecular hydrogen bonding interactions as well as aromatic π–π stacking interactions between the imidazole and benzene rings of neighbouring cations [Cg1···Cg2 = 3.7203 (12)Å; 1-x, 1-y, 1-z, where Cg1 and Cg2 are centroids of the imidazole (N1/C10/N2/C11/C12) and benzene (C1–C6) rings, respectively, Fig. 2].

### Experimental

A mixture of the ionic liquid 1-(2-aminoethyl)-3-methylimidazolium hexafluorophosphate (4 mmol) and 2,4-dichlorobenzaldehyde (3 mmol) was stirred for 4 h at room temperature under solvent-free conditions. After completion ofthe reaction, ethanol (30 ml) was added to the reaction mixture, filtered off the solid product and washed with cold ethanol. The crude product was purified by recrystallization in ethanol/ethyl acetate(3:1 *v*/*v*). Single crystals suitable for X-ray diffraction were obtained by slow evaporation of an ethyl acetate solution of the complex at room temperature.

### Refinement

All H atoms were located in a difference Fourier maps and were refined as ridingatoms: C—H = 0.95–0.99 Å and with *U*_iso_(H) = 1.2 *U*_eq_(C).

### Crystal data

**Table:**

C_13_H_14_Cl_2_N_3_^+^·PF_6_^−^	*Z* = 2
*M**_r_* = 428.14	*F*(000) = 432
Triclinic, *P*1	*D*_x_ = 1.702 Mg m^−^^3^
Hall symbol: -P 1	Mo *K*α radiation, λ = 0.71073 Å
*a* = 8.3465 (13) Å	Cell parameters from 5376 reflections
*b* = 10.1419 (16) Å	θ = 2.7–27.0°
*c* = 11.0310 (17) Å	µ = 0.55 mm^−^^1^
α = 78.899 (2)°	*T* = 173 K
β = 76.523 (2)°	Block, colorless
γ = 67.834 (2)°	0.32 × 0.24 × 0.21 mm
*V* = 835.3 (2) Å^3^	

### Data collection

**Table:**

Bruker SMART CCD area-detector diffractometer	3566 independent reflections
Radiation source: fine-focus sealed tube	3151 reflections with *I* > 2σ(*I*)
graphite	*R*_int_ = 0.0000
φ and ω scans	θ_max_ = 27.0°, θ_min_ = 1.9°
Absorption correction: multi-scan (*SADABS*; Sheldrick, 1996)	*h* = −10→10
*T*_min_ = 0.844, *T*_max_ = 0.894	*k* = −12→12
3566 measured reflections	*l* = −14→13

### Refinement

**Table:**

Refinement on *F*^2^	Primary atom site location: structure-invariant direct methods
Least-squares matrix: full	Secondary atom site location: difference Fourier map
*R*[*F*^2^ > 2σ(*F*^2^)] = 0.031	Hydrogen site location: inferred from neighbouring sites
*wR*(*F*^2^) = 0.093	H-atom parameters constrained
*S* = 1.10	*w* = 1/[σ^2^(*F*_o_^2^) + (0.047*P*)^2^ + 0.3955*P*] where *P* = (*F*_o_^2^ + 2*F*_c_^2^)/3
3566 reflections	(Δ/σ)_max_ = 0.001
227 parameters	Δρ_max_ = 0.25 e Å^−^^3^
0 restraints	Δρ_min_ = −0.34 e Å^−^^3^

### Special details

**Table:**

Geometry. All esds (except the esd in the dihedral angle between two l.s. planes) are estimated using the full covariance matrix. The cell esds are taken into account individually in the estimation of esds in distances, angles and torsion angles; correlations between esds in cell parameters are only used when they are defined by crystal symmetry. An approximate (isotropic) treatment of cell esds is used for estimating esds involving l.s. planes.
Refinement. Refinement of *F*^2^ against ALL reflections. The weighted *R*-factor *wR* and goodness of fit *S* are based on *F*^2^, conventional *R*-factors *R* are based on *F*, with *F* set to zero for negative *F*^2^. The threshold expression of *F*^2^ > σ(*F*^2^) is used only for calculating *R*-factors(gt) *etc*. and is not relevant to the choice of reflections for refinement. *R*-factors based on *F*^2^ are statistically about twice as large as those based on *F*, and *R*- factors based on ALL data will be even larger.

### Fractional atomic coordinates and isotropic or equivalent isotropic displacement parameters (Å^2^)

**Table:**

	*x*	*y*	*z*	*U*_iso_*/*U*_eq_	
C1	0.3066 (2)	0.34376 (17)	0.75848 (15)	0.0220 (3)	
C2	0.3555 (2)	0.30042 (17)	0.87666 (15)	0.0227 (3)	
C3	0.4986 (2)	0.17904 (17)	0.90058 (15)	0.0241 (3)	
H3	0.5296	0.1513	0.9817	0.029*	
C4	0.5944 (2)	0.09983 (17)	0.80205 (16)	0.0240 (3)	
C5	0.5499 (2)	0.13684 (18)	0.68374 (16)	0.0270 (3)	
H5	0.6167	0.0796	0.6180	0.032*	
C6	0.4071 (2)	0.25808 (18)	0.66287 (15)	0.0257 (3)	
H6	0.3761	0.2841	0.5818	0.031*	
C7	0.1562 (2)	0.47512 (17)	0.73301 (15)	0.0237 (3)	
H7	0.0901	0.5323	0.7989	0.028*	
C8	−0.0351 (2)	0.64629 (18)	0.61084 (16)	0.0283 (4)	
H8A	−0.0911	0.6807	0.6939	0.034*	
H8B	−0.1235	0.6282	0.5765	0.034*	
C9	0.0251 (2)	0.76060 (18)	0.52316 (16)	0.0297 (4)	
H9A	−0.0739	0.8533	0.5247	0.036*	
H9B	0.1211	0.7719	0.5539	0.036*	
C10	0.0061 (2)	0.79621 (17)	0.29722 (15)	0.0244 (3)	
H10	−0.1003	0.8765	0.3020	0.029*	
C11	0.2433 (2)	0.62263 (18)	0.22472 (17)	0.0295 (4)	
H11	0.3313	0.5609	0.1688	0.035*	
C12	0.2369 (2)	0.61583 (18)	0.34869 (17)	0.0285 (4)	
H12	0.3197	0.5483	0.3968	0.034*	
C13	0.0537 (3)	0.7880 (2)	0.06825 (17)	0.0368 (4)	
H13A	−0.0631	0.8627	0.0741	0.055*	
H13B	0.0539	0.7081	0.0304	0.055*	
H13C	0.1410	0.8276	0.0161	0.055*	
Cl1	0.23667 (6)	0.39837 (5)	1.00213 (4)	0.03149 (12)	
Cl2	0.78059 (6)	−0.04833 (5)	0.82692 (4)	0.03387 (13)	
F1	0.44475 (17)	0.19348 (17)	0.31806 (17)	0.0655 (4)	
F2	0.58081 (16)	0.32801 (12)	0.36186 (12)	0.0436 (3)	
F3	0.70635 (18)	0.08681 (13)	0.38373 (11)	0.0492 (3)	
F4	0.82315 (14)	0.21845 (14)	0.22679 (11)	0.0412 (3)	
F5	0.68843 (16)	0.08228 (12)	0.18313 (11)	0.0423 (3)	
F6	0.56548 (19)	0.32368 (14)	0.15939 (13)	0.0555 (4)	
N1	0.08759 (18)	0.72527 (14)	0.39294 (13)	0.0243 (3)	
N2	0.09752 (19)	0.73655 (15)	0.19419 (13)	0.0262 (3)	
N3	0.11363 (19)	0.51306 (15)	0.62537 (13)	0.0263 (3)	
P1	0.63280 (5)	0.20636 (5)	0.27210 (4)	0.02671 (12)	

### Atomic displacement parameters (Å^2^)

**Table:**

	*U*^11^	*U*^22^	*U*^33^	*U*^12^	*U*^13^	*U*^23^
C1	0.0223 (7)	0.0219 (8)	0.0212 (7)	−0.0075 (6)	−0.0039 (6)	−0.0016 (6)
C2	0.0239 (7)	0.0235 (8)	0.0204 (7)	−0.0080 (6)	−0.0019 (6)	−0.0049 (6)
C3	0.0259 (8)	0.0241 (8)	0.0227 (8)	−0.0088 (6)	−0.0070 (6)	−0.0002 (6)
C4	0.0216 (7)	0.0178 (7)	0.0298 (8)	−0.0042 (6)	−0.0047 (6)	−0.0016 (6)
C5	0.0284 (8)	0.0242 (8)	0.0259 (8)	−0.0062 (7)	−0.0016 (6)	−0.0070 (6)
C6	0.0280 (8)	0.0272 (8)	0.0204 (7)	−0.0075 (7)	−0.0048 (6)	−0.0027 (6)
C7	0.0235 (8)	0.0223 (8)	0.0233 (8)	−0.0057 (6)	−0.0038 (6)	−0.0031 (6)
C8	0.0278 (8)	0.0279 (9)	0.0233 (8)	−0.0011 (7)	−0.0079 (6)	−0.0032 (6)
C9	0.0379 (9)	0.0236 (8)	0.0257 (8)	−0.0038 (7)	−0.0120 (7)	−0.0050 (6)
C10	0.0256 (8)	0.0197 (7)	0.0260 (8)	−0.0053 (6)	−0.0062 (6)	−0.0019 (6)
C11	0.0259 (8)	0.0229 (8)	0.0361 (9)	−0.0073 (7)	0.0005 (7)	−0.0053 (7)
C12	0.0217 (8)	0.0223 (8)	0.0373 (9)	−0.0034 (6)	−0.0060 (7)	−0.0013 (7)
C13	0.0491 (11)	0.0397 (10)	0.0220 (8)	−0.0165 (9)	−0.0065 (8)	−0.0017 (7)
Cl1	0.0345 (2)	0.0313 (2)	0.0223 (2)	−0.00086 (17)	−0.00639 (16)	−0.00879 (16)
Cl2	0.0295 (2)	0.0252 (2)	0.0392 (2)	0.00147 (16)	−0.00988 (17)	−0.00417 (17)
F1	0.0325 (7)	0.0723 (10)	0.0967 (12)	−0.0247 (7)	0.0080 (7)	−0.0315 (9)
F2	0.0411 (6)	0.0347 (6)	0.0488 (7)	−0.0033 (5)	−0.0004 (5)	−0.0202 (5)
F3	0.0676 (9)	0.0348 (6)	0.0269 (6)	−0.0002 (6)	−0.0056 (5)	−0.0010 (5)
F4	0.0279 (6)	0.0566 (7)	0.0404 (6)	−0.0150 (5)	0.0001 (5)	−0.0158 (5)
F5	0.0519 (7)	0.0373 (6)	0.0390 (6)	−0.0120 (5)	−0.0087 (5)	−0.0140 (5)
F6	0.0626 (9)	0.0399 (7)	0.0548 (8)	0.0017 (6)	−0.0332 (7)	0.0033 (6)
N1	0.0253 (7)	0.0194 (6)	0.0269 (7)	−0.0046 (5)	−0.0080 (5)	−0.0027 (5)
N2	0.0282 (7)	0.0245 (7)	0.0249 (7)	−0.0092 (6)	−0.0028 (5)	−0.0028 (5)
N3	0.0277 (7)	0.0235 (7)	0.0244 (7)	−0.0038 (6)	−0.0072 (5)	−0.0024 (5)
P1	0.0213 (2)	0.0254 (2)	0.0292 (2)	−0.00174 (17)	−0.00511 (16)	−0.00594 (17)

### Geometric parameters (Å, °)

**Table:**

C1—C2	1.397 (2)	C9—H9B	0.9900
C1—C6	1.401 (2)	C10—N2	1.324 (2)
C1—C7	1.479 (2)	C10—N1	1.330 (2)
C2—C3	1.387 (2)	C10—H10	0.9500
C2—Cl1	1.7414 (16)	C11—C12	1.345 (3)
C3—C4	1.383 (2)	C11—N2	1.382 (2)
C3—H3	0.9500	C11—H11	0.9500
C4—C5	1.383 (2)	C12—N1	1.380 (2)
C4—Cl2	1.7394 (16)	C12—H12	0.9500
C5—C6	1.378 (2)	C13—N2	1.466 (2)
C5—H5	0.9500	C13—H13A	0.9800
C6—H6	0.9500	C13—H13B	0.9800
C7—N3	1.266 (2)	C13—H13C	0.9800
C7—H7	0.9500	F1—P1	1.5798 (13)
C8—N3	1.462 (2)	F2—P1	1.5991 (12)
C8—C9	1.521 (3)	F3—P1	1.6059 (12)
C8—H8A	0.9900	F4—P1	1.5945 (12)
C8—H8B	0.9900	F5—P1	1.6077 (12)
C9—N1	1.474 (2)	F6—P1	1.5903 (13)
C9—H9A	0.9900		
			
C2—C1—C6	117.32 (15)	N1—C10—H10	125.6
C2—C1—C7	122.19 (14)	C12—C11—N2	106.95 (15)
C6—C1—C7	120.49 (14)	C12—C11—H11	126.5
C3—C2—C1	122.51 (15)	N2—C11—H11	126.5
C3—C2—Cl1	116.75 (12)	C11—C12—N1	107.24 (15)
C1—C2—Cl1	120.74 (12)	C11—C12—H12	126.4
C4—C3—C2	117.56 (15)	N1—C12—H12	126.4
C4—C3—H3	121.2	N2—C13—H13A	109.5
C2—C3—H3	121.2	N2—C13—H13B	109.5
C3—C4—C5	122.21 (15)	H13A—C13—H13B	109.5
C3—C4—Cl2	118.75 (13)	N2—C13—H13C	109.5
C5—C4—Cl2	119.02 (13)	H13A—C13—H13C	109.5
C6—C5—C4	118.87 (15)	H13B—C13—H13C	109.5
C6—C5—H5	120.6	C10—N1—C12	108.39 (14)
C4—C5—H5	120.6	C10—N1—C9	124.53 (14)
C5—C6—C1	121.51 (15)	C12—N1—C9	127.08 (14)
C5—C6—H6	119.2	C10—N2—C11	108.67 (14)
C1—C6—H6	119.2	C10—N2—C13	125.07 (15)
N3—C7—C1	121.45 (15)	C11—N2—C13	126.20 (15)
N3—C7—H7	119.3	C7—N3—C8	116.53 (14)
C1—C7—H7	119.3	F1—P1—F6	91.02 (9)
N3—C8—C9	110.67 (14)	F1—P1—F4	179.50 (9)
N3—C8—H8A	109.5	F6—P1—F4	89.48 (8)
C9—C8—H8A	109.5	F1—P1—F2	90.12 (7)
N3—C8—H8B	109.5	F6—P1—F2	91.08 (7)
C9—C8—H8B	109.5	F4—P1—F2	89.81 (6)
H8A—C8—H8B	108.1	F1—P1—F3	90.46 (9)
N1—C9—C8	112.47 (14)	F6—P1—F3	178.38 (8)
N1—C9—H9A	109.1	F4—P1—F3	89.04 (7)
C8—C9—H9A	109.1	F2—P1—F3	89.56 (7)
N1—C9—H9B	109.1	F1—P1—F5	90.54 (7)
C8—C9—H9B	109.1	F6—P1—F5	89.83 (7)
H9A—C9—H9B	107.8	F4—P1—F5	89.52 (6)
N2—C10—N1	108.75 (14)	F2—P1—F5	178.86 (7)
N2—C10—H10	125.6	F3—P1—F5	89.51 (7)
			
C6—C1—C2—C3	0.9 (2)	N3—C8—C9—N1	67.10 (18)
C7—C1—C2—C3	−178.59 (15)	N2—C11—C12—N1	0.05 (19)
C6—C1—C2—Cl1	−179.03 (12)	N2—C10—N1—C12	−0.11 (18)
C7—C1—C2—Cl1	1.5 (2)	N2—C10—N1—C9	179.91 (15)
C1—C2—C3—C4	0.0 (2)	C11—C12—N1—C10	0.03 (19)
Cl1—C2—C3—C4	179.95 (12)	C11—C12—N1—C9	−179.99 (16)
C2—C3—C4—C5	−1.1 (2)	C8—C9—N1—C10	111.90 (18)
C2—C3—C4—Cl2	177.09 (12)	C8—C9—N1—C12	−68.1 (2)
C3—C4—C5—C6	1.1 (3)	N1—C10—N2—C11	0.14 (18)
Cl2—C4—C5—C6	−177.02 (13)	N1—C10—N2—C13	−177.43 (15)
C4—C5—C6—C1	−0.1 (3)	C12—C11—N2—C10	−0.12 (19)
C2—C1—C6—C5	−0.8 (2)	C12—C11—N2—C13	177.41 (16)
C7—C1—C6—C5	178.67 (15)	C1—C7—N3—C8	−178.82 (14)
C2—C1—C7—N3	179.95 (16)	C9—C8—N3—C7	113.18 (17)
C6—C1—C7—N3	0.5 (2)		

### Hydrogen-bond geometry (Å, °)

**Table:**

*D*—H···*A*	*D*—H	H···*A*	*D*···*A*	*D*—H···*A*
C7—H7···Cl1	0.95	2.69	3.0846 (17)	106
C12—H12···F2	0.95	2.48	3.239 (2)	137
C5—H5···F3	0.95	2.51	3.324 (2)	143
C11—H11···F6	0.95	2.46	3.275 (2)	143
C10—H10···F3^i^	0.95	2.33	3.203 (2)	152
C10—H10···F5^i^	0.95	2.54	3.373 (2)	147
C13—H13C···F5^ii^	0.98	2.54	3.464 (2)	158

Symmetry codes: (i) *x*−1, *y*+1, *z*; (ii) −*x*+1, −*y*+1, −*z*.
